# Membrane Localization of RNase Y Is Important for Global Gene Expression in *Bacillus subtilis*

**DOI:** 10.3390/ijms25158537

**Published:** 2024-08-05

**Authors:** Soumaya Laalami, Marina Cavaiuolo, Jacques Oberto, Harald Putzer

**Affiliations:** 1 Expression Génétique Microbienne, CNRS, Institut de Biologie Physico-Chimique, Université Paris Cité, 75005 Paris, France; laalami@ibpc.fr (S.L.);; 2 Laboratory for Food Safety, SBCL Unit, University Paris Est, ANSES, 94701 Maisons-Alfort, France; 3 Integrative Biology of the Cell (I2BC), CEA, CNRS, Université Paris-Saclay, 91198 Gif-sur-Yvette, France; jacques.oberto@i2bc.paris-saclay.fr

**Keywords:** *Bacillus subtilis*, RNA degradation, RNase Y, membrane localization

## Abstract

RNase Y is a key endoribonuclease that regulates global mRNA turnover and processing in *Bacillus subtilis* and likely many other bacteria. This enzyme is anchored to the cell membrane, creating a pseudo-compartmentalization that aligns with its role in initiating the decay of mRNAs primarily translated at the cell periphery. However, the reasons behind and the consequences of RNase Y’s membrane attachment remain largely unknown. In our study, we examined a strain expressing wild-type levels of a cytoplasmic form of RNase Y from its chromosomal locus. This strain exhibits a slow-growth phenotype, similar to that of an RNase Y null mutant. Genome-wide data reveal a significant impact on the expression of hundreds of genes. While certain RNA substrates clearly depend on RNase Y’s membrane attachment, others do not. We observed no correlation between mRNA stabilization in the mutant strains and the cellular location or function of the encoded proteins. Interestingly, the Y-complex, a specificity factor for RNase Y, also appears also recognize the cytoplasmic form of the enzyme, restoring wild-type levels of the corresponding transcripts. We propose that membrane attachment of RNase Y is crucial for its functional interaction with many coding and non-coding RNAs, limiting the cleavage of specific substrates, and potentially avoiding unfavorable competition with other ribonucleases like RNase J, which shares a similar evolutionarily conserved cleavage specificity.

## 1. Introduction

The instability of messenger RNA is fundamental to the control of gene expression in all organisms. This instability allows bacteria to quickly adapt to changing environments, produce the appropriate amount of a given protein, and recycle ribonucleotides for new RNA synthesis. To maximize bacterial competitiveness, mRNA degradation must be tightly regulated and the most efficient way to achieve this is by controlling the steps that initiate mRNA decay. The important roles of the key endoribonucleases RNase Y (*B. subtilis*) and RNase E (*E. coli*) in generating short-lived decay intermediates are well established. This can be summarized as “different enzymes, similar strategies” [1].

In *B. subtilis*, RNase Y cleaves its substrates with a specificity similar to RNase E, targeting UA-rich single-stranded regions, preferably on 5′ monophosphorylated substrates. The depletion of RNase Y increases global mRNA stability [2]. This RNA decay pathway affects the levels of the majority of transcripts in *B. subtilis* [3,4,5] and *S. pyogenes* [6] but has a more limited effect in *S. aureus* [7]. Identifying RNase Y cleavage sites in *B. subtilis* and other Gram-positive organisms confirms a preference for UA-rich single-stranded sequences, often flanked by secondary structures [2,7,8,9,10,11].

Degradosome-like complexes based on RNase Y have been proposed [12,13], but they are generally unstable without cross-linking, except possibly under certain stress conditions [14]. Whether other ribonucleases can form meaningful interactions with RNase Y in vivo remains an open question [1,15,16,17]. Currently, in *B. subtilis*, the most significant effect on RNase Y activity in vivo is mediated by three small proteins: YaaT, YlbF, and YmcA [18]. These proteins can stably bind to each other, forming the so-called Y-complex [19,20], which is required for the efficient maturation of many operon mRNAs and determines the abundance of certain riboswitch RNAs [9,21]. Since the Y-complex does not affect all RNase Y targets, it can be considered a specificity factor for this globally acting endoribonuclease [9].

RNase Y is tethered to the inner side of the membrane by a single-pass N-terminal helix ([22], referred to as YmdA in this reference). This pseudo-compartmentalization is a feature shared with *E. coli* RNase E [23,24] and is consistent with the predominant distribution of translating ribosomes along the cell periphery [25,26]. Available data provide some insight into the importance of membrane tethering for enzymes that initiate RNA decay. In *E. coli*, detaching RNase E from the inner membrane results in a global slow-down of RNA degradation and an increased turnover of ribosome-free transcripts [27]. In *S. aureus*, where RNase Y plays a minor role in initiating mRNA decay compared with *B. subtilis*, releasing the enzyme from the membrane slows growth but does not significantly alter its activity profile [7].

In *B. subtilis*, RNase Y moves rapidly along the membrane in the form of dynamic short-lived foci. Upon transcription arrest, these foci become more abundant and increase in size, suggesting they do not represent the enzyme’s most active form [21]. This contrasts with the similar formation of foci by RNase E in *E. coli*, which depends on the presence of RNA substrates [24]. The YaaT component of the Y-complex also localizes to the cell periphery [28] in a manner dependent on the presence of RNase Y [9]. Mutations in the Y-complex have an even stronger effect than RNA depletion on increasing the size and number of RNase Y foci at the membrane. This suggests that the Y-complex may modify RNase Y activity by shifting the assembly status of the enzyme toward more active smaller membrane complexes [21]. Thus, tethering RNase Y to the membrane appears to be important not only for spatial confinement but also for controlling the activity of the enzyme. However, whether this localization plays a role in global RNA metabolism in *B. subtilis* remains unknown.

Here, we analyze the significance of the attachment of RNase Y to the membrane by examining the effects of an *rny*∆TMD mutation on RNA degradation. This allele expresses an RNase Y mutant lacking the N-terminal transmembrane domain at physiological levels from the wild-type chromosomal locus. This cytoplasmic version of the enzyme, which is uniformly distributed throughout the cell, results in slower growth and extensive changes in the levels of hundreds of transcripts. We identified a range of RNAs that require RNase Y tethering to the membrane and others that do not. Our observations indicate that the subcellular localization of wild-type RNase Y does not correlate with its activity toward specific classes or types of RNA substrates. We discuss the functional implications and rationale for the membrane attachment of RNase Y.

## 2. Results

### 2.1. Effects of Cytoplasmic RNase Y on Cell Morphology and Fitness

**Figure 1:**
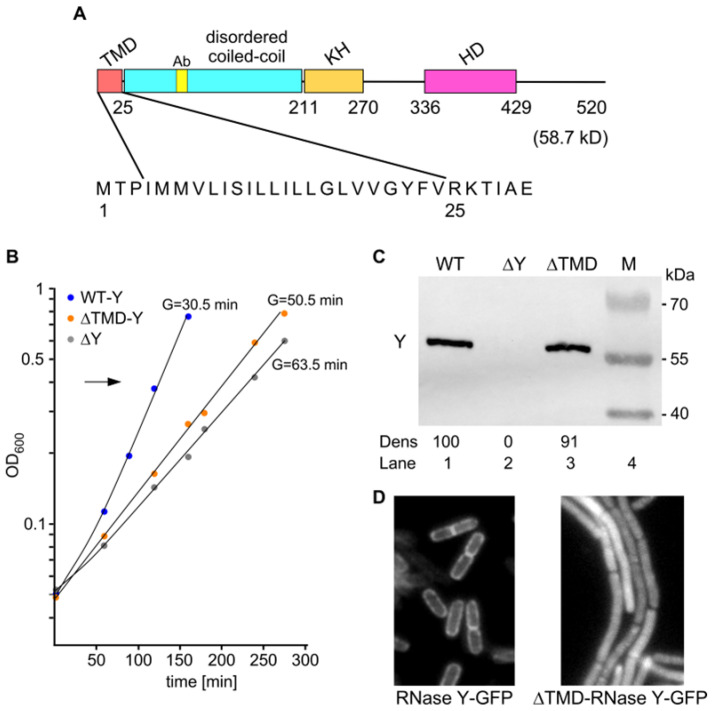
Structure and expression of ∆TMD-RNase Y; physiological parameters of the mutant strain. (**A**). Domains composing *B. subtilis* RNase Y (520 aa) include an N-terminal transmembrane domain (TMD, aa 1–25), followed by a large region predicted to be disordered (aa ~30–210), an RNA binding KH domain (aa 211–270), a metal-chelating HD domain (aa 336–429) containing the conserved His/Asp motif required for RNase activity [2,22,30,31,32] and a C-terminal domain of unknown function (aa 429-520). Ab indicates the position of the 12 aa peptide used for monoclonal antibody production. The hydrophobic 21 amino acid stretch composing the RNase Y transmembrane region is shown below the domain structure scheme. The cytoplasmic version of RNase Y (∆TMD-RNase Y) expressed in strain SSB574 lacks aa 2 to 24. (**B**). Growth curves in LB medium at 37 °C. Strains expressing wild-type RNase Y (SSB507), cytoplasmic ∆TMD-RNase Y (SSB574), or no RNase Y (SSB508) were harvested at OD_600_ ~0.4 (arrow). (**C**). Expression of RNase Y and ∆TMD-RNase Y as well as the absence of RNase Y in the different strains was analyzed by Western blot. (**D**) ∆TMD-RNase Y localizes in the cytoplasm. Wild-type RNase Y-sfGFP (SSB2048) and ∆TMD-RNase Y-sfGFP (SSB2066) expression was analyzed by epifluorescence microscopy in cells grown to the mid-log phase.

In order to study how the membrane localization of RNase Y might affect its biological activity in vivo, we constructed a strain expressing a cytoplasmic form of the enzyme. *B. subtilis* RNase Y is composed of a large ~200 aa N-terminal region predicted to be disordered, followed by an RNA binding KH domain and a metal-chelating HD domain required for RNase activity (Figure 1A). The first 25 amino acids at the N-terminal extremity comprise a transmembrane domain (TMD), a stretch of 21 predominantly hydrophobic residues beginning at residue 4 (Figure 1A). There are no charged residues at the N terminus, but the hydrophobic region is immediately followed by two basic residues (R, K). According to the positive inside rule of von Heinje (1992), these features are strongly suggestive of a single transmembrane region with the C-terminal part of the protein located in the cytosol. In agreement, an RNase Y-GFP fusion protein lacking the aforementioned 21 amino acids (∆TMD) clearly localizes to the cytoplasm, in contrast to wild-type RNase Y-GFP (Figure 1D).

In a complex medium (LB), a strain lacking RNase Y (∆*rny*, SSB508) grew with a >2-fold longer generation time compared with the wild-type strain: 63.5 min versus 30.5 min, respectively. A similar growth defect was observed in a strain exclusively expressing the cytoplasmic form of RNase Y (Figure 1B). In this strain, ∆TMD-RNase Y was present at a level equivalent to that observed for wild-type RNase Y (Figure 1C), as expected, since the 63 nt (21 aa) deletion was introduced in the wild-type *rny* gene without any additional alteration. Since the enzyme retains all structural domains besides the membrane anchor and is active in this form in vitro [2], the localization of RNase Y itself seems to be important for its biological activity and hence to support efficient growth. The ∆TMD strain grows as chains while wild-type cells grow as single or dividing cells (Figure 1D). Nevertheless, cytoplasmic RNase Y can restore the severe defects in cell morphology of a ∆*rny* strain that, under the same growth conditions, forms spirals interspersed with long chains [29].

### 2.2. Cytoplasmic Localization of RNase Y Affects Gene Expression on a Genome-Wide Scale

**Figure 2:**
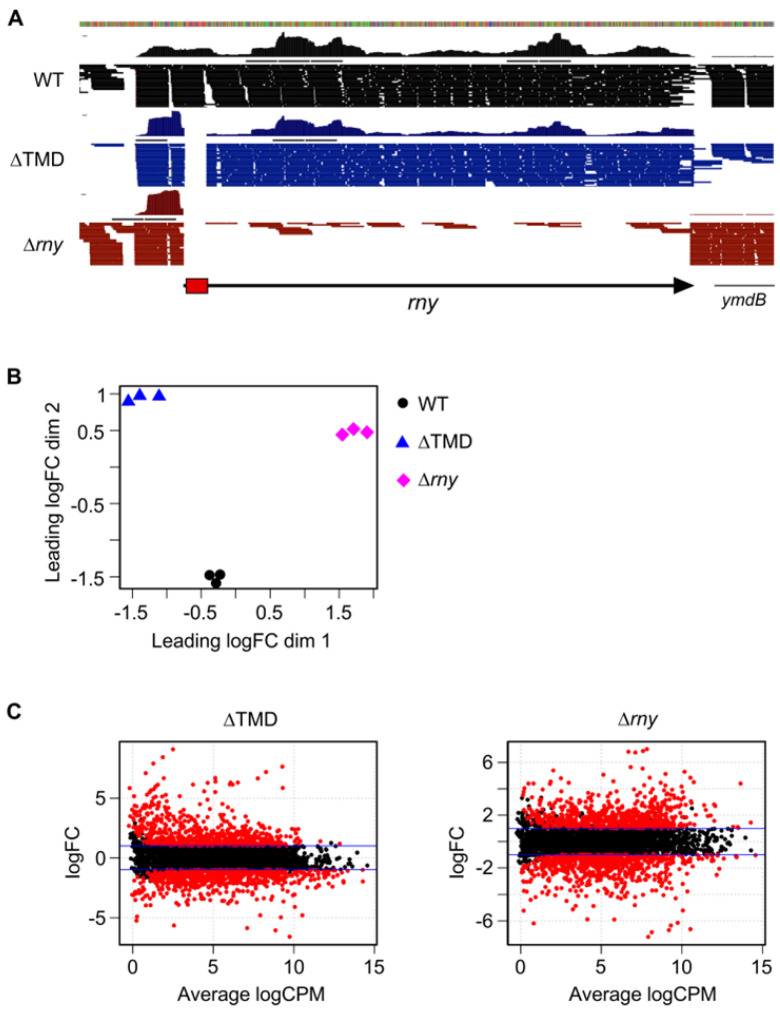
Global effects on the transcriptome in strains expressing ∆TMD—RNase Y or no RNase Y. (**A**). Browser view of RNA-Seq reads of the *rny* locus of the wild-type and mutant strains expressing the ∆TMD version of RNase Y or lack the *rny* ORF. The red box indicates the gene sequences coding for the N-terminal TMD domain. Reads from a single replicate of each strain are shown as representative examples. (**B**). Multidimensional scaling (MDS) plot of WT, ∆TMD, and ∆*rny* triplicate libraries. Distance between samples is based on the leading log2 fold-change (FC) of the top 500 most differentially regulated genes. (**C**). MA plots of differentially expressed genes (red dots) at FDR ≤ 0.05 identified in the two mutant strains compared to WT.

To better apprehend the role of RNase Y membrane localization on global gene expression, we sequenced and compared the transcriptomes of the wild-type, an RNase Y null mutant strain (∆rny), and a strain expressing a cytoplasmic version of RNase Y (∆TMD) using stranded RNA-Seq. A total of ~145 million raw sequence reads was obtained from the analysis of 9 samples, with an average per sample of ~16 million reads (Table S1) mapped to the *B. subtilis* genome. The absence of reads corresponding to the entire rny ORF or the 63 nt of the TMD region confirmed the integrity of the ∆*rny* and ∆TMD strains, respectively (Figure 2A).

Differential gene expression between all strains was explored by measuring the distance of gene expression values between any pair of samples. The data sets, filtered for poorly or not expressed genes, consisted of 4105 RNA features including open reading frames and untranslated regions (UTRs, riboswitches, and small RNAs) derived from the chromosome and retrieved from Subtiwiki [33]. On a multidimensional scaling (MDS) plot (Figure 2B), the triplicates within each group (strain) clustered closely, indicating similar gene expression levels and consistency between biological replicates. By contrast, both the ∆*rny* and the ∆TMD strains were clearly separated from the WT samples. This suggested that localization to the membrane via the TMD is important for the normal function of RNase Y. These observations are corroborated by the MA plots of the expression data (Figure 2C) that relate the ratio of level counts for each RNA feature between WT and mutant strains against the average level counts for each feature from all libraries. We applied a cutoff of FC ≥ 2 and ≤ 2 at an FDR ≤ 0.05 to call for differentially expressed gene (DEG) features (red dots above and below the blue line in Figure 2C), and we categorized them into functional groups according to Gene Ontology (GO) annotation (Supplementary Tables S2 and S3).

Compared to WT, the ∆*rny* and the ∆TMD-RNase Y expressing strains had similar numbers of DEGs (Table 1): 712 and 665 upregulated features and 751 and 652 downregulated features, respectively. An increased mRNA level in the absence of a given RNase is often an indication of direct action of the enzyme on the substrate RNA, while downregulated RNAs are generally caused by indirect effects, as observed in most RNase-related transcriptome studies. The expression of cytoplasmic ∆TMD-RNase Y might be expected to increase the cleavage of natural RNase Y substrates. Indeed, 72 RNAs that are upregulated in the ∆*rny* mutant have significantly lower levels in the ∆TMD strain compared with the WT strain (Table S3). However, the majority of the downregulated RNAs in the ∆TMD strain (462 out of 652) are likely not genuine RNase Y substrates because they are also present at lower levels in the ∆*rny* mutant. In both mutant strains, most of the upregulated genes belonged to the GO categories «undefined», «unknown», and «general function prediction», 41% (293 in total) and 42.6% (292 in total) in the ∆*rny* and ∆TMD-RNase Y strains, respectively (Table S2). There were several functional categories in which significantly more genes were induced in the ∆TMD strain compared with the ∆*rny* strain: “Carbohydrate transport and metabolism” (53 vs. 24 transcripts), “Lipid transport and metabolism” (27 vs. 14 mRNAs), and “Energy production and conversion” (24 vs. 13 mRNAs). About 30% of the transcripts with significantly increased levels in the ∆TMD strain (203) compared with a WT strain were also induced in the ∆*rny* knock-out strain. Of those, 45 mRNAs were prophage-related (SBbeta and PBSX) and, together with the remaining 158 transcripts, are good candidates for RNase Y substrates that require the localization of the enzyme at the membrane for efficient cleavage (Table S4). An analysis of 55 riboswitch, 5′ leader sequences and other non-coding RNAs showed that more than half (29) have higher levels in the ∆*rny* strain, and 11 are also induced in the presence of cytoplasmic RNase Y (Table S5). There is no general pattern as to which type of non-coding RNA is an RNase Y substrate and/or requires membrane localization of the enzyme, e.g., T-box or guanine riboswitches can be found in all categories (Table S5).

The detection of transcripts that are induced only in the ∆TMD strain but not in the ∆*rny* strain is intriguing (Table S6). One possibility could be that cytoplasmic RNase Y masks cleavage sites prone to be cleaved by other endoribonucleases (see below).

### 2.3. Modulation of Specific Transcript Levels by Cytoplasmic RNase Y

We performed Northern blots of specific transcripts to confirm some of the RNA-Seq data and to analyze more precisely if and how cytoplasmic RNase Y can alter transcription patterns. We focused on transcripts whose intracellular levels were significantly affected in one or both mutant strains, with a preference for known RNase Y substrates and/or mRNAs encoding proteins susceptible to influencing the activity of RNase Y. The RNA substrates were divided into two categories, one that requires and one that is insensitive to membrane localization of RNase Y.

#### 2.3.1. RNA Substrates that Require Membrane Localization of RNase Y

**Figure 3:**
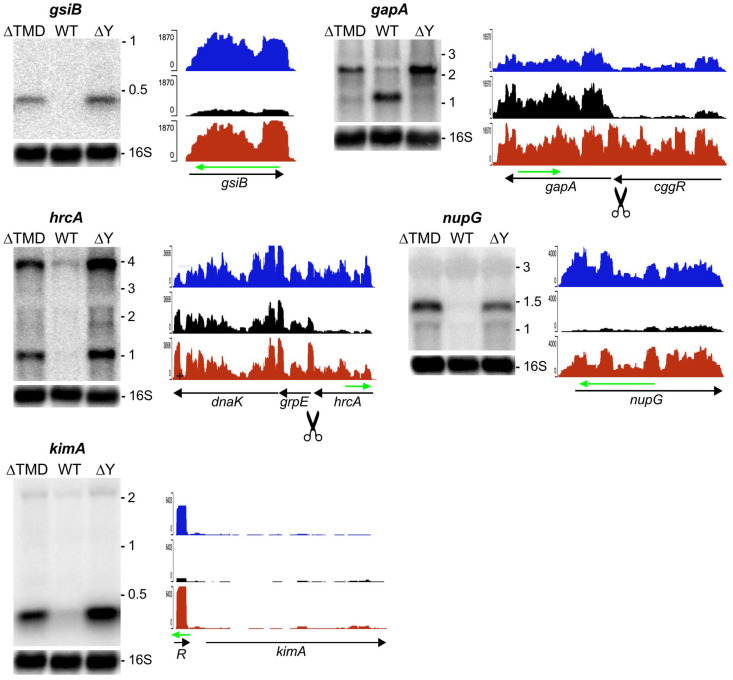
Examples of coding and non-coding RNAs requiring RNase Y membrane attachment for maintaining wild-type transcript patterns. The (**left panels**) show a Northern analysis of total RNA isolated from WT, ∆TMD, and ∆*rny* strains. The blots were hybridized to specific riboprobes complementary to the regions indicated by a green arrow above the indicated gene. The RNAseq profiles (**right panels**) for the ∆TMD (blue), WT (black), and ∆Y (red) strains are at the same scale for a given gene. When known, the identified RNase Y cleavage site is indicated by a scissors symbol.

Transcripts that require RNase Y to be anchored at the membrane for cleavage should be upregulated not only in the ∆*rny* mutant but also in a strain expressing the cytoplasmic version of the nuclease. In Figure 3, several examples of transcripts that have elevated levels in both mutant strains compared with a wild-type strain are shown.

The *gsiB* gene encodes a small general stress protein. The monocistronic 0.4 kb transcript is transcribed from a σ^B^ promoter under various stress conditions, like glucose starvation or heat shock [34]. The very low *gsiB* mRNA level present in exponentially growing WT cultures used here is strongly increased in the absence of RNase Y and to almost the same level in the strain expressing cytoplasmic RNase Y (see *gsiB* in Figure 3). This indicates that cytoplasmic ∆TMD-RNase Y cannot replace the membrane-tethered wild-type enzyme to act on the *gsiB* transcript.

Regulatory processing by RNase Y in the *cggR* mRNA 3′ end uncouples *gapA* and *cggR* expression [12]. In the absence of RNase Y, the 2.2 kb *gapA-cggR* transcript accumulates (Figure 3). As judged by the ratio of reads corresponding to the *cggR* and *gapA* ORFs, some cleavage still occurs in the ∆TMD strain, but cytoplasmic RNase Y is clearly less efficient in this processing event (Figure 3, *gapA*).

A cleavage with a similar outcome occurs in the intergenic region between *hrcA* and *grpE*. It uncouples the expression of the transcriptional repressor HrcA from the other components of the *dnaK* heat shock operon that it controls [35]. As expected for cultures grown at 37 °C, the transcript levels from this operon are very low in the wild-type strain. However, the levels of both the monocistronic *hrcA* and the tricistronic *hrcA-grpE-dnaK* transcripts are strongly increased in the RNase Y knock-out strain (Figure 3, *hcrA*), highlighting the importance of the mRNA processing step in the DnaK-mediated heat shock response. The localization of RNase Y at the membrane was critically important, as a strain expressing the enzymatically active ∆TMD-RNase Y had the same elevated transcript pattern as the knock-out mutant (Figure 3, *hrcA*).

Another example is the *nupG* mRNA encoding a purine nucleoside transporter (Figure 3, *nupG*). Transcription is under the control of a guanine-dependent riboswitch (G-box) [36] that is strongly induced in both the ∆*rny* and the ∆TMD strains (Table S5). Whether the cleavage of the riboswitch is sufficient to maintain *nupG* mRNA wild-type levels or also requires cleavage by membrane-bound RNase Y within the *nupG* ORF is unknown.

RNase Y is known to initiate the degradation of many riboswitches [2,5], and here, we found that several of those sensitive to RNase Y also require the membrane localization of the enzyme. This is the case for the cyclic di-AMP riboswitch that controls the expression of the high-affinity K^+^ transporter KimA (Figure 3, [37,38]). By contrast, the second cyclic di-AMP riboswitch upstream of the *ktrA* gene does not appear to be an RNase Y substrate. Again, there is no rule as to which class of riboswitches is susceptible to RNase Y cleavage and/or a requirement for its localization at the membrane (confer Table S5).

#### 2.3.2. RNA Substrates not Sensitive to RNase Y Localization

**Figure 4:**
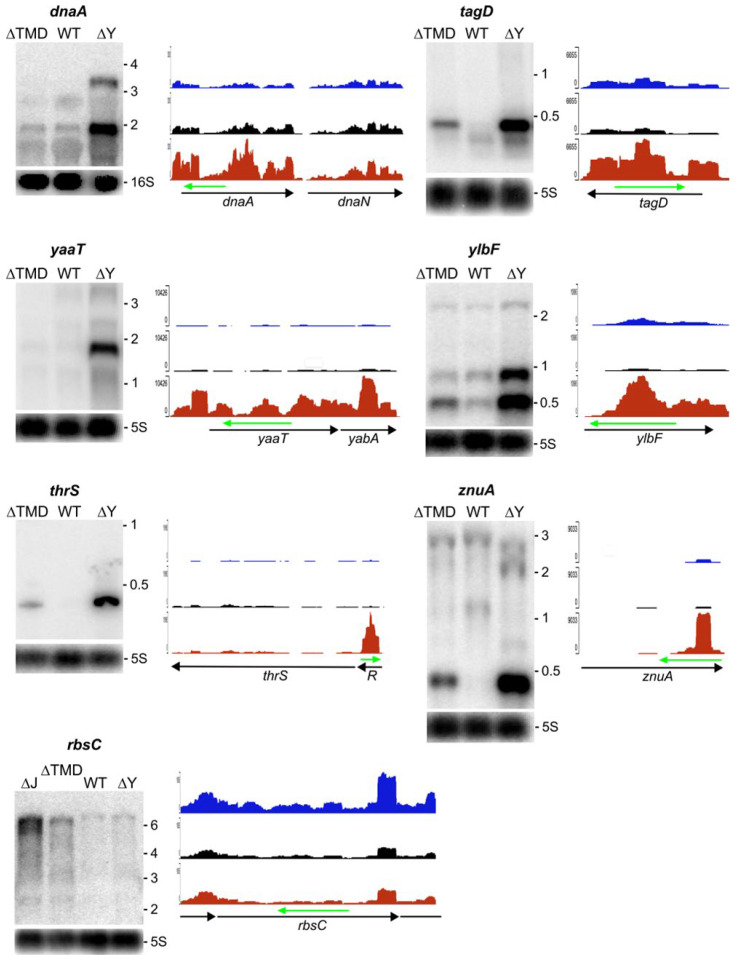
Examples of coding and non-coding RNAs sensitive to both the membrane attached and the cytoplasmic form of RNase Y. The (**left panels**) show a Northern analysis of total RNA isolated from WT, ∆TMD, and ∆*rny* strains. The blots were hybridized to specific riboprobes complementary to the regions, indicated by a green arrow above the indicated gene. The RNAseq profiles (**right panels**) for the ∆TMD (blue), WT (black), and ∆Y (red) strains are at the same scale for a given gene. For the *rbsC* gene, the Northern analysis also includes RNA isolated from the ∆*rnjA*/∆*rnjB* double mutant expressing no RNase J.

Transcripts that do not require RNase Y to be tethered to the membrane for cleavage should be present at the same or a similar level in the ∆TMD strain expressing cytoplasmic RNase Y when compared to the wild-type strain. Roughly 60–70% of the transcripts increased in the ∆*rny* mutant fall into this category; many of these transcripts show actually lower levels in the ∆TMD strain suggesting a more efficient cleavage (Table S3). We analyzed several examples by Northern blot (Figure 4), some of which were previously shown to be a direct target of RNase Y [5]. The expression of the entire *tagD* and the divergent *tagA* operon as well as the *tagO* gene, all of which are crucial for cell wall synthesis, is strongly induced in the ∆*rny* strain (Table S3). All of these transcripts are present at close to wild-type levels in the ∆TMD strain. As shown for *tagD*, the 0.4 kb mRNA is strongly reduced compared with the ∆*rny* strain but remains slightly more abundant than that in the wild-type strain (Figure 4).

The essential *dnaA-dnaN* operon encoding the replication initiator protein (DnaA) and the β-subunit of DNA polymerase III (the beta clamp, DnaN) is transcribed into mono- and bicistronic mRNAs (~1.6 and ~3 kb) that are strongly stabilized in the absence of RNase Y [5], Figure 4). In this case, cytoplasmic ∆TMD-RNase Y is able to maintain exactly the transcript pattern and levels observed in the wild-type strain (Figure 4, *dnaA*).

The so-called Y-complex composed of three small proteins YaaT, YlbF, and YmcA [20] was shown to bind to and modify RNase Y activity and assembly status in vivo [9,18,21]. The transcript levels of all three genes are upregulated in the ∆*rny* strain (Figure 4, *yaaT*, *ylbF*, and Table S3), as observed previously [5,9]. Since individual Y-complex mutations alone affect the transcript levels of the remaining Y-complex proteins [9], it is likely that the Y-complex autoregulates its expression through its interaction with RNase Y. As shown in Figure 4, RNase Y does not need to be at the membrane to maintain wild-type levels of the *yaaT* and *ylbF* transcripts (see also discussion).

As mentioned above, many riboswitches and non-coding RNAs do not require RNase Y to be at the membrane to initiate their degradation. One example is the T-box riboswitch controlling threonyl-tRNA synthetase (*thrS*) expression. The prematurely terminated riboswitch RNA (~0.3 kb) is present only at very low levels in the ∆TMD strain, similar to the wild-type strain (Figure 4, *thrS*).

A peculiar non-coding RNA is transcribed from an internal sigma A promoter in the *znuA* (*adcA*) gene encoding a zinc-binding lipo-protein [39]. Comprised of the 400 3′ proximal nucleotides, it is only detected in the absence of RNase Y. Again, cytoplasmic RNase Y can efficiently replace the wild-type enzyme (Figure 4, *znuA*).

There are a number of transcripts that are only induced in the presence of cytoplasmic RNase Y but not in the absence of the enzyme (Table S6). There is no straightforward explanation for this observation. We have previously shown that RNase Y and RNase J1/J2 (and *E. coli* RNase E) can cleave the *thrS* leader sequence at the same site [40,41], suggesting convergent evolution toward a common enzymatic activity. However, cleavage efficiency can be very different in vitro and in vivo. For example, RNase J1 cleaves the *thrS* leader much better in vitro than RNase Y, yet in vivo cleavage is mediated almost exclusively by RNase Y [40]. Since both enzymes RNase Y and RNase J can obviously recognize but not necessarily cleave with the same efficiency in a given sequence context, normal cleavage patterns could be perturbed by the increased concentration of one or the other enzyme. The expression of ∆TMD-RNase Y even at wild-type levels increases the local concentration of RNase Y in the cytoplasm and could lead to increased binding and masking of cleavage sites on RNAs that are normally a substrate for RNase J. An example of such a configuration is the *rbsC* transcript encoding a ribose ABC transporter [42]. The level of the major polycistronic ribose operon transcript including *rbsC* (~6.3 kb) is increased in the ∆TMD but not the ∆rny strain (Figure 4, *rbsC*). If cytoplasmic RNase Y acts by masking a potential RNase J cleavage site in this mRNA, the transcript level should be equal or higher in an RNase J mutant. This is exactly what we observed in the RNase J1/J2 double mutant (Figure 4, *rbsC*).

## 3. Discussion

In this study, we showed that cytoplasmic expression of RNase Y has a profound effect on the physiology and the global transcription pattern in *B. subtilis*. In LB media, the strain with the *rny*∆TMD allele grows 1.7-fold more slowly than the wild-type control, which is similar to the twofold effect observed for the ∆*rny* null mutant. The pleiotropic changes induced by the ∆TMD mutation do not allow us to pinpoint a single cause for the growth defect. An obvious reason for the observed phenotype might reside in an altered activity of the mutant enzyme. However, we have previously measured comparable enzyme activities in vitro for ∆TMD-RNase Y and the wild-type enzyme bound to multilamellar vesicles prepared from *B. subtilis* native lipids [2,43]. Moreover, the wild-type and the cytoplasmic versions of RNase Y are both expressed from the chromosomal wild-type locus and are present in equivalent amounts in the cell. We thus favor the hypothesis that differences observed for many potential RNA substrates are not due to the enzyme activity per se but rather to other factors: (i) altered accessibility of the RNA substrate by the cytoplasmic enzyme and (ii) a potential difference in the interaction of the two RNase Y forms with auxiliary factors like the Y-complex (see below).

Among the transcripts that were upregulated in the ∆*rny* strain and that are potential direct RNase Y targets, 203 also had higher levels in the presence of cytoplasmic RNase Y. We concentrated on the 158 transcripts that were not prophage-related. A Northern analysis of several of them confirmed that cleavage/maturation of the RNA requires or is strongly stimulated by RNase Y tethered to the membrane. We previously showed that *E. coli* RNase E can quite efficiently replace RNase Y in *B. subtilis* and that the single most important parameter to efficient complementation was the requirement for RNase E to localize to the membrane [40]. It is noteworthy that about one-third (48) of the 158 transcripts highlighted to depend on RNase Y membrane localization also required RNase E to be localized at the membrane for restoring wild-type levels in a ∆*rny* mutant. Even though the experimental conditions were similar but not identical in the two studies, this suggests that the accessibility of the substrate to RNase Y could be an important factor for the cleavage of a considerable number of RNA substrates. An interesting example is the HrcA repressor protein that controls nine class I heat shock genes organized in two operons. The complex regulation of this regulon, which also involves the activation of HrcA by one of the regulated genes [35,44], is not fully understood. The strong upregulation of the *hrcA* mRNA in the presence of cytoplasmic RNase Y nevertheless suggests an important role for the membrane localization of RNase Y in the heat shock response of *B. subtilis.*

The conditioned interaction with partner proteins could be another reason why certain RNA substrates require membrane localization of RNase Y. The Y-complex (YaaT, YlbF, and YmcA) that localizes to the membrane in a manner dependent on RNase Y has previously been shown to act as an auxiliary factor required for the efficient cleavage of many polycistronic mRNAs [9]. It can shift the assembly status of membrane-bound, higher-order structures of RNase Y toward fewer and smaller complexes, which are likely the more active form of the enzyme [21]. Referring to the available data from the Y-complex transcriptome study [9], we have checked whether the five transcripts that required membrane localization of RNase Y (Figure 3) might also be sensitive to Y-complex mutations. With the exception of the *kimA* (*ydaO*) riboswitch, which was not even sensitive to the absence of RNase Y in that study, the other transcripts all required the Y-complex for maintaining wild-type transcript levels. This supports the hypothesis that in some cases, RNase Y membrane attachment is important for cleavage because the binding of the Y-complex might require the higher-order structures of RNase Y found at the membrane. When comparing the data on a larger scale, we found that only about 20% of transcripts preferentially cleaved by membrane-tethered RNase Y are also upregulated in Y-complex mutant strains. However, one has to consider that the compared data sets were derived from different strains: *B. subtilis* 168 used in our study and the less-domesticated *B. subtilis* 3610 in the Y-complex study. Together with the fact that many of the transcripts were present at very low levels (Tables S3 and S4 in [9]) and that membrane-dependent cleavage is rarely an all-or-nothing effect, this might also explain the limited accordance.

More than two-thirds of the transcripts that are upregulated in the ∆*rny* strain are maintained at near wild-type levels in the strain expressing cytoplasmic RNase Y. They include a number of essential genes involved in initiation of DNA replication (e.g., *dnaA*, Figure 4) and biosynthesis of cell wall teichoic acids (e.g., *tagABDGH*, Figure 4). For several of them, RNase Y has been shown to act directly by modulating the half-life of the respective transcripts [5]. The deregulation of these genes has been linked to aberrant cell morphology [45,46]. Restoring their correct expression could explain the almost-normal cell shape observed in the ∆TMD strain compared to the RNase Y null mutant.

Interestingly, maintaining wild-type levels of the Y-complex transcripts can also be achieved by cytoplasmic RNase Y. Since the putative autoregulation of the Y-complex proteins likely occurs via RNase Y cleavage of the respective mRNAs, this implies that the Y-complex should also be able to interact with ∆TMD-RNase Y. It is unclear which oligomeric state RNase Y might have in the cell when expressed as a cytoplasmic form of the enzyme. The N-terminal intrinsically unstructured domain of RNase Y by itself (~200 aa) lacking the TMD can form dimers that contribute to the formation of ∆TMD-RNase Y dimeric forms [32]. However, when overexpressed and purified from *E. coli*, ∆TMD-RNase Y is always present in various amounts of high-molecular-weight oligomers in addition to the dimeric form [2,32]. Further studies are required to determine whether the function of the Y-complex involves interactions with an RNase Y dimer or requires higher-order structures like those present at the membrane [21].

We found no obvious reason or common attributes that could indicate why a given RNA substrate would require RNase Y to be attached to the membrane or not. Nevertheless, we determined the presence of transmembrane domains for all protein-encoding gene products but found no significant link between RNase Y membrane localization and a potential preference for RNA substrates encoding membrane proteins (Tables S3–S6). This is in agreement with a previous study that found no strong bias for the localization of membrane protein-encoding transcripts at the cell periphery [47]. In *B. subtilis*, transcription and translation occur predominantly in separate functional domains with ribosomes distributed at the inner membrane and the cell poles [25,26]. It thus makes sense that the mRNA degradation machinery is localized at the membrane where it can monitor suboptimal translation and initiate mRNA decay to maintain efficient gene expression. At the same time, unrelated observations from *E. coli* suggest that localization of the decay-initiating RNase at the membrane might not necessarily require a substrate RNA to diffuse to the cell periphery in order to be cleaved. Indeed, an artificially membrane-attached transcriptional antiterminator protein (i.e., *E. coli* BglG) is capable of interacting fast enough with its chromosomally encoded nascent mRNA target sequence to promote transcriptional read-through [48].

The subcellular organization of the major decay-initiating endoribonucleases RNases Y and E in bacteria can be achieved by different strategies, like membrane anchoring [21,24] and the formation of bacterial ribonucleoprotein bodies (BR-bodies, [49]). This pseudo-compartmentalization is generally advantageous for cell growth, but its importance varies in different organisms. In *E. coli*, attachment of RNase E to the inner membrane assures optimal rates of global mRNA degradation and protects ribosome-free transcripts from increased turnover [27]. In *S. aureus*, the detachment of RNase Y from the membrane slows growth but does not alter the number and cleavage profiles of the roughly hundred RNase Y substrates identified in this organism [7]. In *B. subtilis*, where RNase Y has a much more important role in global mRNA turnover [5], the cytoplasmic expression of RNase Y influenced the expression of hundreds of genes. These include three types of transcripts: (i) those that require membrane localization of RNase Y; (ii) those that do not, including RNAs downregulated compared to the wild-type strain, most likely due to the more efficient access of the enzyme to the substrate; and (iii) potential RNA substrates that are specifically upregulated in the presence of ∆TMD-RNase Y. The number of 295 transcripts might be overestimated, as some values for the ∆*rny* strain are close to the 2× cut-off (Table S6). Nevertheless, this could highlight another potentially important aspect of pseudo-compartmentalization, which is competition between enzymes with similar activity. We have previously shown that RNase J1/J2 and RNase Y have evolved toward an evolutionarily conserved endo-nucleolytic activity [40,50]. The individual effects of RNase J1 and J2 mutations on the transcriptome have been analyzed [51], but the extent of endo-nucleolytic cleavages mediated by RNase J1/J2 is unknown. A plausible explanation for the specific induction of transcripts in the presence of ∆TMD-RNase Y could be that cytoplasmic RNase Y can recognize but not efficiently cleave certain RNase J cleavage sites. The increased level of the *rbsC* mRNA in the ∆J mutant and the ∆TMD strain tends to confirm this hypothesis. However, a more detailed analysis of this and other potential RNA substrates is required to validate this competition model. Some bacteria, like many firmicutes and delta-proteobacteria, have all three RNases—RNases Y, J, and E [1]; among them are many pathogenic organisms, like *Clostridia* and *Listeria*, in which ribonucleases play important roles in virulence and pathogenicity [52,53]. In these organisms, homeostatic mechanisms, including the subcellular location of major RNA decay enzymes with similar specificity, are likely important to optimize RNA metabolism.

## 4. Materials and Methods

### 4.1. Bacterial Strains and Growth Conditions

The *B. subtilis* strains used in this work (Table 2) are derivatives of strain SSB1002; a wild-type laboratory stock strain derived from strain 168. *E. coli* strain JM109 [54] was used for plasmid construction. *B. subtilis* strain SSB507 is wild-type for *rny* and contains the empty pDR160T vector integrated at *amyE.* SSB508 is derived from SSB507, in which the *rny* ORF has been replaced in frame with that of chloramphenicol acetyltransferase (651 nt) from the *S. aureus* plasmid pC194 [55], as described [21]. SSB574 carries the same *rny* deletion as SSB508 and a xylose-inducible copy of the *rny* gene lacking the 5′ terminal transmembrane domain (aa 2–24) integrated at *amyE* (plasmid pHMD40). The primers used in this study are listed in Table 3. *B. subtilis* and *E. coli* strains were grown at 37 °C in an LB medium with aeration. The expression of cytoplasmic (∆TMD) RNase Y was induced by the addition of 50 mM xylose to the medium. When required, the following antibiotics were added to the medium: chloramphenicol (5 μg/mL) and spectinomycin (100 μg/mL).

Strains SSB2048 and SSB2066 express RNase Y-sfGFP and ∆TMD-RNase Y-sfGFP from the native *rny* locus, as described [21]. Briefly, they were constructed by markerless allelic replacement using the thermoexcisable plasmid pMAD [56], and epifluorescence images were taken from mid-log cultures with a 100x oil objective on a Zeiss AxioImager M1, as described [21].

### 4.2. Plasmid Constructs

*pDR160T*. Ectopic integration vector (at *amyE*) expressing inserted genes from the xylose-inducible Psweet promoter [57]. The original vector was modified to contain the *rnjA* 3′ transcription terminator in order to terminate transcripts transcribed from the xylose inducible promoter [40].

*pHMD40*. Plasmid pHMD40 contains a mutated *rny* gene lacking amino acids 2 to 24 (∆TMD), encoding a cytoplasmic version of RNase Y under the control of the xylose-inducible promoter and the wild-type *rny* Shine–Dalgarno sequence. A 1.5 kb PCR fragment (oligonucleotides HP1827-HP1696) was cleaved with PacI and BamHI and ligated into the respective sites of plasmid pDR160T.

### 4.3. Epi-Fluorescence Microscopy

GFP fluorescent images were taken with the Zeiss Axio Imager M1 microscope equipped with an AxioCam MRm camera (Zeiss, Oberkochen, Germany) using filter set 10 (Zeiss). For the visualization of cells from exponentially growing cultures, overnight cultures in LB medium were diluted to OD600 ∼0.1 and grown at 37◦C in fresh LB medium for at least three generations. Cells were mounted on 1% (*w*/*v*) agarose pads, and images were acquired with an AxioCam camera MRm (Zeiss) using a 1.3 NA 100× oil objective.

### 4.4. Northern Blot

RNA blot analysis was carried out using 5 μg of total RNA separated on 0.8 or 1.2% formaldehyde agarose gels. The RNA was transferred by capillary blotting to a Hybond N+ membrane (GE Healthcare, Chicago, IL, USA) and UV cross-linked at 120 mJ cm^−2^ for 1 min. Hybridization to specific RNA probes was carried out as described previously [40].

### 4.5. Western Blot

For Western blot analysis, a monoclonal RNase Y antibody directed against a 12 aa peptide (residues 79–90) was used to detect RNase Y in 20 μg of protein extract, as described previously [58].

### 4.6. Transcriptome Analysis

The RNAseq analysis (library preparation and sequencing) was performed at the iGE3 Genomics Platform of the University of Geneva (https://ige3.genomics.unige.ch, accessed on 28 July 2024). RNA samples were treated with the Ribo-Zero Kit for Bacteria (Illumina) to remove ribosomal RNA (rRNAs). Multiplex RNA-Seq libraries were prepared with the Illumina TruSeq Stranded Total RNA Sample Preparation and sequenced (HiSeq2000). The complete genome sequence of the *Bacillus subtilis* subsp. *subtilis* str. 168 (NCBI accession number for chromosome: NC 000964.3) and its annotation were retrieved from NCBI (https://www.ncbi.nlm.nih.gov/genome/?term=Bacillus±subtilis (accessed on 28 July 2024)) and Subtiwiki [33]. Reads processing, mapping, and differential gene expression analysis were performed according to [59]. Briefly, single-end 100-nt reads were mapped to the reference genome using the Burrows–Wheeler Alignment tool (BWA) and allowing soft-clipping in the alignment parameters (BWA MEM) [60]. The analysis of the mappings used Samtools for the processing, sorting, and indexing of the alignment files; BEDtools for computing coverage; and the Integrative Genomics Viewer (IGV) browser for displaying the alignment files [60,61,62]. Reads counts were normalized either as reads per million (RPM) or transcripts for million (TPM). Differential gene expression analysis between strains was performed with the EdgeR package [63] using the Trimmed Mean of M-values (TMM) scaling factor as the normalization method. Gene features were considered significantly up- or downregulated in the mutants versus the WT samples if the log2 fold-change (FC) ratio was ≥1 or ≤–1 (or FC ratio ≥2 or ≤−2) and the *p*-value adjusted for the multiple testing False Discovery Rate (FDR) calculated using the Benjamini–Hochberg (BH) method in EdgeR was equal or lower than 5% (FDR ≤ 0.05). Multi-dimensional scaling (MDS) and MA plots (visualizing measurement differences between two samples by transforming the data onto M (log ratio) and A (mean average scales) were generated, respectively, through the ‘plotMDS.dge’ and the plotSmear functions of the EdgeR package (version 3.14.0).

## 5. Conclusions

In this study, we explored the critical role of RNase Y’s membrane localization in *B. subtilis*. When we detached RNase Y from the membrane, we observed a severe impact on bacterial growth, mirroring the effects seen in an RNase Y null mutant. Interestingly, when RNase Y is expressed in the cytoplasm, it alters the expression of hundreds of genes.

We discovered that while many RNA substrates clearly depend on RNase Y’s membrane attachment, others do not. However, we found no obvious correlation between these classes of transcripts and the cellular location or function of their encoded proteins, including membrane proteins.

Our findings suggest that RNase Y’s membrane attachment may be important not only for spatial confinement but also for forming higher-order structures that interact with other specificity factors like the Y-complex. Although the Y-complex can function with RNase Y detached from the membrane, the oligomeric state of the cytoplasmic enzyme remains unknown.

Notably, some RNA substrates are specifically upregulated when RNase Y is cytoplasmic, which we attribute to competition between RNase Y and RNase J for similar recognition sites. This evolutionarily conserved cleavage specificity among RNases Y, E, and J underpins the rationale for the pseudo-compartmentalization of these enzymes. In organisms that possess all three types of RNases, it seems crucial to spatially confine them to maintain specific cleavage activities and optimize RNA metabolism.

## Figures and Tables

**Table 1:** Number of up- and downregulated transcripts with a fold-change (FC) of ≥2 or ≤−2 (FDR ≤ 0.05) in mutant strains compared with WT.

Strain, Relevant Genotype	No. Upregulated Transcripts	No. Downregulated Transcripts
SSB574, ∆TMD-RNase Y	665	652
SSB508, ∆*rny*	712	751

**Table 2:** *B. subtilis* strains used in this study.

*B. subtilis* Strain	Relevant Genotype	Reference
SSB1002	Wild-type strain	Lab stock
SSB507	*∆amyE::pDR160T*	This work
SSB508	*∆rny::cat*, *∆amyE::pDR160T*	This work
SSB574	*∆rny::cat*, *∆amyE::pHMD40*	This work

**Table 3:** Oligonucleotides used in this study.

Oligonucleotide	Sequence 5′-3′
HP1696	GACTCGAGCCGTAGAGTATGCAAAATAAAGGATCCTATC
HP1827	AATGATTAATTAACAACAACCAAGTTCATAGCAAGAGGAGGTGAAAGTATGCGTAAAACCATTGCCGAAGCG

## Data Availability

Raw sequencing data have been deposited at the NCBI Sequence Read Archive, accession number (SRA) BioProject: PRJNA675751 (https://dataview.ncbi.nlm.nih.gov/object/PRJNA675751?reviewer=5ppknrt8j0179c3i311ihto055), accessed on 28 July 2024.

## Supplementary Materials

The following supporting information can be downloaded at: https://www.mdpi.com/article/10.3390/ijms25158537/s1.
